# Synthesis and Biocompatibility Studies of New Iminodiacetic Acid Derivatives

**DOI:** 10.3390/molecules22122265

**Published:** 2017-12-18

**Authors:** Magdalena Markowicz-Piasecka, Piotr Dębski, Elżbieta Mikiciuk-Olasik, Joanna Sikora

**Affiliations:** 1 Laboratory of Bioanalysis, Department of Pharmaceutical Chemistry, Drug Analysis and Radiopharmacy, Medical University of Lodz, ul. Muszyńskiego1, 90-151 Lodz, Poland; joanna.sikora@umed.lodz.pl; 2 Students Research Group, Laboratory of Bioanalysis, Department of Pharmaceutical Chemistry, Drug Analysis and Radiopharmacy, Medical University of Lodz, ul. Muszyńskiego 1, 90-151 Lodz, Poland; piotrdebski.93@gmail.com; 3 Department of Pharmaceutical Chemistry, Drug Analysis and Radiopharmacy, Medical University of Lodz, ul. Muszyńskiego 1, 90-151 Lodz, Poland; elzbieta.mikiciuk-olasik@umed.lodz.pl

**Keywords:** iminodiacetic acid, radiopharmaceuticals, biocompatibility, haemostasis

## Abstract

Background: Iminodiacetic acid (IDA) derivatives can be used as ligands to form complexes with technetium, with potential application as hepatobiliary diagnostic agents. The aim of this study was to synthesize five novel IDA derivatives and to compare their effects on plasma haemostasis with clinically approved ligands for technetium complexation. Methods: The influence of synthesized IDA derivatives on plasma haemostasis was evaluated spectrophotometrically by clot formation and lysis test (CL-test), coagulation assay, Prothrombin Time and Activated Partial Tromboplastin Time. The effects of the tested compounds on erythrocytes were assessed using haemolysis assays, microscopy and flow cytometry studies. Results: Despite their significant influence on the kinetic parameters of the process of clot formation and fibrinolysis, the tested ligands, at potential diagnostic concentrations, did not alter the overall potential of clot formation and lysis (CL_AUC_). At potential diagnostic concentrations (0.4 μmol/mL) all the tested compounds showed no adverse effects on the membranes of RBCs (Red Blood Cells). Conclusion: IDA derivatives with methoxy substituents in aromatic ring, exert multidirectional effects on plasma haemostasis and should be considered safe as their significant impacts were mostly observed at 4 μmol/mL, which is about 10-fold higher than the theoretical plasma concentrations of these compounds.

## 1. Introduction

Any novel chemical substance (e.g., drugs, drug carriers, contrast agents used for imaging) or biomedical material (e.g., implants, sutures, glues) must reach their biocompatibility before being put into clinical use. A substance or material is considered biocompatible when it is introduced into the body and induces normal and expected biological responses without causing adverse effects to the human organism [1]. From a regulatory stance, biocompatibility is measured by a series of tests that are used to determine the potential toxicity from contact with the medical devices or result from the products within the body [2]. Among the tests included in evaluating biocompatibility, which are spelled out in ISO 10993, are cytotoxicity, sensitization, irritation/intracutaneous reactivity, acute systemic toxicity, pyrogenecity, subacute/subchronic toxicity, genetic toxicology, implantation, and hemocompatibility studies [2].

The typical way of administrating diagnostic agents (including both contrast agents (CAs) and radiopharmaceuticals) is intravenously. Ideally, these compounds should only exert a minimum effect on the blood and vascular endothelium. Among radiopharmaceuticals, used for diagnostic and therapeutic purposes, the most common radioisotope is Tc, especially its metastable nuclear isomer ^99m^Tc. The results of the latest literature review suggest that technetium due to its quadrupole moment and large spin can become a promising candidate for the EPR (Electron Paramagnetic Resonance) spectroscopy and MRI studies [3,4,5]. Mancini et al. [4] claim that ^99^Tc-NMR spectroscopy can be used as a powerful spectroscopic technique used for characterization of Tc complexes.

In the case of radiopharmaceuticals used for diagnostic purposes the acceptance of ^99m^Tc is so rapid that there is little time to investigate how the body deals with radiopharmaceuticals labelled with this radionuclide. There are studies evaluating the effects of radiopharmaceuticals, including their binding, on blood constituents which have shown that these findings cannot be easily compared. In addition, the authors state that in nuclear medicine, it is essential to understand the interactions of radiopharmaceuticals with blood constituents not only in order to anticipate the behavior in vivo of radiotrace but also to predict its toxicity [6,7].

Iminodiacetic acid derivatives after radiolabelling with technetium (^99m^Tc), can be used as radiopharmaceuticals (e.g., ^99m^Tc-bromotriethyl-IDA (mebrofenin)) during cholescintigraphy [8,9]. Following intravenous administration, ^99m^Tc complexes with IDA derivatives, such as mebrofenin, bind to plasma proteins (mainly albumins). In the liver, in the space of Disse, labelled complex dissociates from the proteins and enters the hepatocyte by a mechanism similar to that of serum bilirubin. It has been shown that polypeptide transporters transporting organic anions (OATPs) are involved in the uptake of IDA derivatives. These proteins are located on the side-cell membrane of hepatocytes. In the transport of mebrofenin, the OATP1B1 and OATP1B3 isoforms take part, however, the affinity of the complex to the isoform OATP1B1 is approximately 1.5 times greater [10,11]. The complexes passage unmetabolized through the hepatocyte and enters the bile canaliculi. In the transport of mebrofenin to the bile polypeptide tubular transporter, namely multidrug resistance protein (MRP2) is involved. Among people with mutation of the gene coding this membrane protein (Dubin-Johnson syndrome) extended systemic exposure to mebrofenin is observed [12].

Apart from above-mentioned ^99m^Tc-bromotriethyl-IDA (mebrofenin) several other IDA derivatives including disofenin (DISIDA), etifenin (EHIDA) and iprofenin (PIPIDA) have been introduced to pharmaceutical market [13].

The results of many previous studies suggest (^99m^)Tc-mebrofenin as the most valuable quantitative radiotracer for functional examination of the liver, suitable for determination of various aspects of its function [14,15]. The influence of unconjugated bilirubin on the uptake of these diagnostic agents is significant from the clinical point of view. Because of bilirubin competitive binding to the protein transporter, its high plasma levels (>8 mg/dL) may interfere with the transport of IDA derivatives with simultaneous increase of their renal elimination. In this respect, mebrofenin is characterized by the highest of all known ligands resistance to high concentration of bilirubin in the blood. Studies have shown that uptake of this complex by hepatocytes was more than 70% for bilirubin at concentration of 20 mg/dL, while in the case of disofenin transport efficiency decreased to 36% at 10 mg/dL. Therefore, in patients with hepatic insufficiency, the use of mebrofenin in scintigraphy allows for a much better visualization of this organ [16].

Increased levels of bilirubin affect not only hepatic uptake of radiopharmaceuticals based on IDA derivatives but also contribute to the changes in the function of coagulation system. For instance, hypercoagulation has been detected among patients with obstructive jaundice. The authors reported a correlation between strength, elasticity and coagulation indices of the clot and increased concentrations of direct bilirubin [17]. Other studies also report a significant positive correlation between high bilirubin concentration and prolonged APTT and PT times, basic markers of coagulation efficacy [18,19,20]. As early as seventies of the previous century it was also found that biliary obstruction can lead to abnormalities of erythrocytes membrane [21]. In our previous study, we found that five IDA derivatives, the ligands for technetium used in cholescintigraphy, exert multi-dimensional effects on the coagulation and fibrinolysis [15]. It was reported that incubation of human plasma with IDA derivatives resulted in a significant increase in the thrombin time, thrombin generation time and initial velocity of fibrin clot formation. Furthermore, these compounds likely altered the structure of the clot, as shown by an increase in the clot stabilization time, which correlated with a delay in the onset of fibrinolysis process [15]. This implies that ligands for Tc complexation, including clinically applied mebrofenin might interfere not only with coagulation process but also with fibrinolysis.

**Figure 1:**
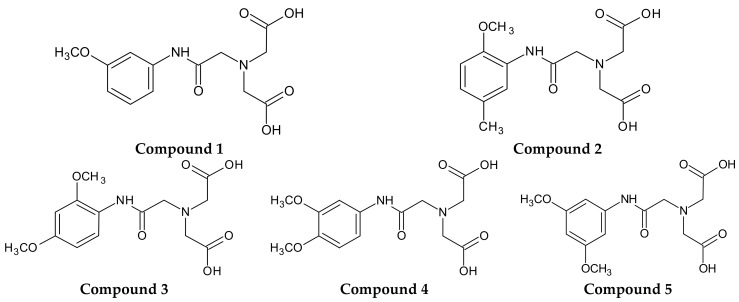
Synthesized derivatives of iminodiacetic acid (IDA).

Therefore, the primary objective of the current investigation was to synthesize novel 5 derivatives of iminodiacetic acid with methoxy substituents in the aromatic ring (Figure 1) and to examine their influence on plasma haemostasis. For this purpose, we established the overall potential of clot formation and fibrinolysis, as well as kinetic parameters of these processes. Additionally, we evaluated the effects of IDA derivatives on the generation of endogenous thrombin and basis coagulology parameters, such as Prothrombin Time (PT), Activated Partial Thromboplastin Time (APTT) and Thrombin Time (TT). Furthermore, we evaluated the influence of the synthesized compounds on the stability of erythrocyte membranes and morphology of RBCs (red blood cells) using microscopy and flow cytometry. Finally, on the basis of conducted studies we draw the conclusion how structure and substituents in aromatic ring of IDA derivatives affect their biological properties with particular emphasis on plasma haemostasis and RBCs morphology. Assignment of the possible biocompatibility of IDA derivatives would be of vital importance for further design and development of potential ligands for cholescintigraphy.

## 2. Results

### 2.1. In Silico Structure-Activity Evaluation

As theoretical calculations of various drug’s parameters are often well correlated with the experimental ones, we decided to use in silico tools in order to elucidate the effects of differently substituted IDA derivatives (Figure 1) on the plasma haemostasis and RBCs membrane permeability. ‘Lipinski’s rule of five’ describes the features of compounds which predispose them to be drugs. They are as follows: appropriate lipophilicity expressed as logP (partition-coefficient between two inmixing phases, n-octanol and water); molecular weight below 500 Da; ten or less hydrogen bond acceptors; and five or less hydrogen bond donors. Molecules which are not in tune with these rules may express poor bioavailability [22,23,24].

The results are presented in Table 1. All tested ligands are characterised by adequate properties according to the ‘Lipinski’s rule of five’—number of violated drug-likeness rules is equal to 0. Generally, all established parameters of different ligands were close to each other.

In Table 2 we summarized the basic physicochemical parameters of iminodiacetic acid derivatives estimated by means of adequate software [25]. Generally, all parameters counted for the ligands were similar to each other.

### 2.2. CL-Test

The effects of IDA derivatives on the overall haemostasis potential and kinetic parameters of clot formation, stabilization and fibrinolysis are presented in Table 3.

Regarding the overall potential of clot formation and lysis (CL_AUC_), we reported that compounds **3** and **5** did not affect this parameter reflecting the general tendency to induce changes in plasma haemostasis. Compound **1** at concentrations of 0.2 μmol/mL to 4.0 μmol/mL statistically significantly decreased the value of CL_AUC_. Only compound **4** at a concentration of 2.0 μmol/mL contributed to an increase in CL_AUC_. The total time of the measured process (T) was statistically significant from the control for the highest concentrations (2.0 and 4.0 μmol/mL) of all the compounds (↑ T).

The analysed compounds exerted significant effects on the kinetic parameters of clot formation. Considering the thrombin time (Tt), which reflects the time elapsed from the addition of exogenous thrombin (0.5 U/mL) to the beginning of clot formation, we found that only compound **5** did not contribute to the alteration of this parameter. Compounds **2** and **4** at the lowest concentration range significantly shortened Tt (↓ Tt), whereas compounds **1** and **3** at 2.0 and 4.0 μmol/mL lengthened Tt (↑ Tt).

When added at 4.0 μmol/mL all IDA derivatives significantly decreased the maximum clotting (↓ Fmax). The value of Fmax was previously shown to be modulated by the fibrinogen level [26]; however, the latter likely did not affect this parameter as all the samples were controlled for their fibrinogen concentrations.

As shown in Table 3, all the examined compounds caused a significant decrease in the initial plasma clotting velocity (↓ Fvo). In the case of compounds **1**, **2**, **3**, and **4**, this alteration was reported for all concentrations, whereas compound **5** induced decrease in Fvo (↓ Fvo) only at the highest concentration (4.0 μmol/mL). The changes in Fvo were reflected by a significant increase in the plasma clotting time (↑ Tf).

Regarding the second phase of the process, i.e., clot stabilization, compounds **1**, **3** and **5** did not change the clot stabilization time (Tc constant). Compound **2** tested at the highest concentration increased Tc, whereas compound **4** statistically significantly increased Tc at 2.0 μmol/mL. Noticeably, the changes in the time of clot stabilization suggest that these ligands may influence the structure of the clot.

The obtained values of maximum lysis (Lmax) corresponded with the values of Fmax which means that previously formed clots were completely lysed. Most changes in the kinetic parameters of the third phase of examined process (fibrinolysis) occurred at the highest concentrations of the examined ligands. Apart from compound **3**, all ligands contributed to statistically significant decrease in the value of the initial velocity of fibrinolysis (↓ Lvo) with concomitant prolongation of fibrinolysis time (↑ Tl). It should be highlighted that all examined compounds, apart from **2**, did not affect the area under the clot formation curve (Sl constant).

### 2.3. Coagulation Assay

In order to determine the effects of iminodiacetic acid derivatives on the process of coagulation after generation of endogenous thrombin, the mixture of calcium chloride (0.015 mmol/mL) and thrombin (0.0375 IU/mL) was added to diluted PPP. The addition of such small amounts of thrombin and calcium chloride did not initiate the coagulation process, but induced a feedback reaction which consequently led to generation of endogenous thrombin and coagulation [15].

**Figure 2:**
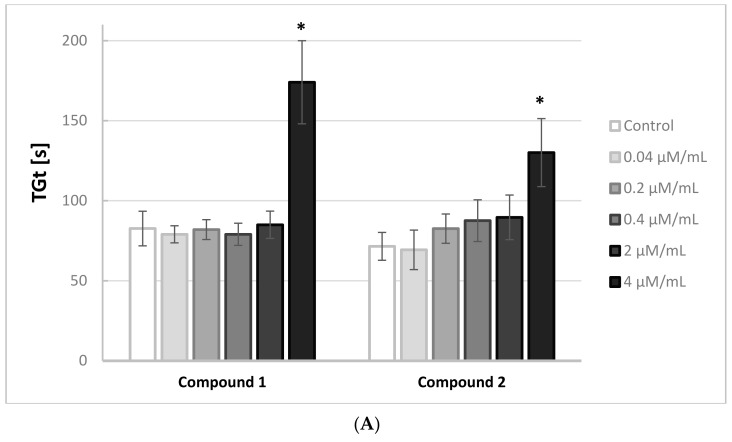

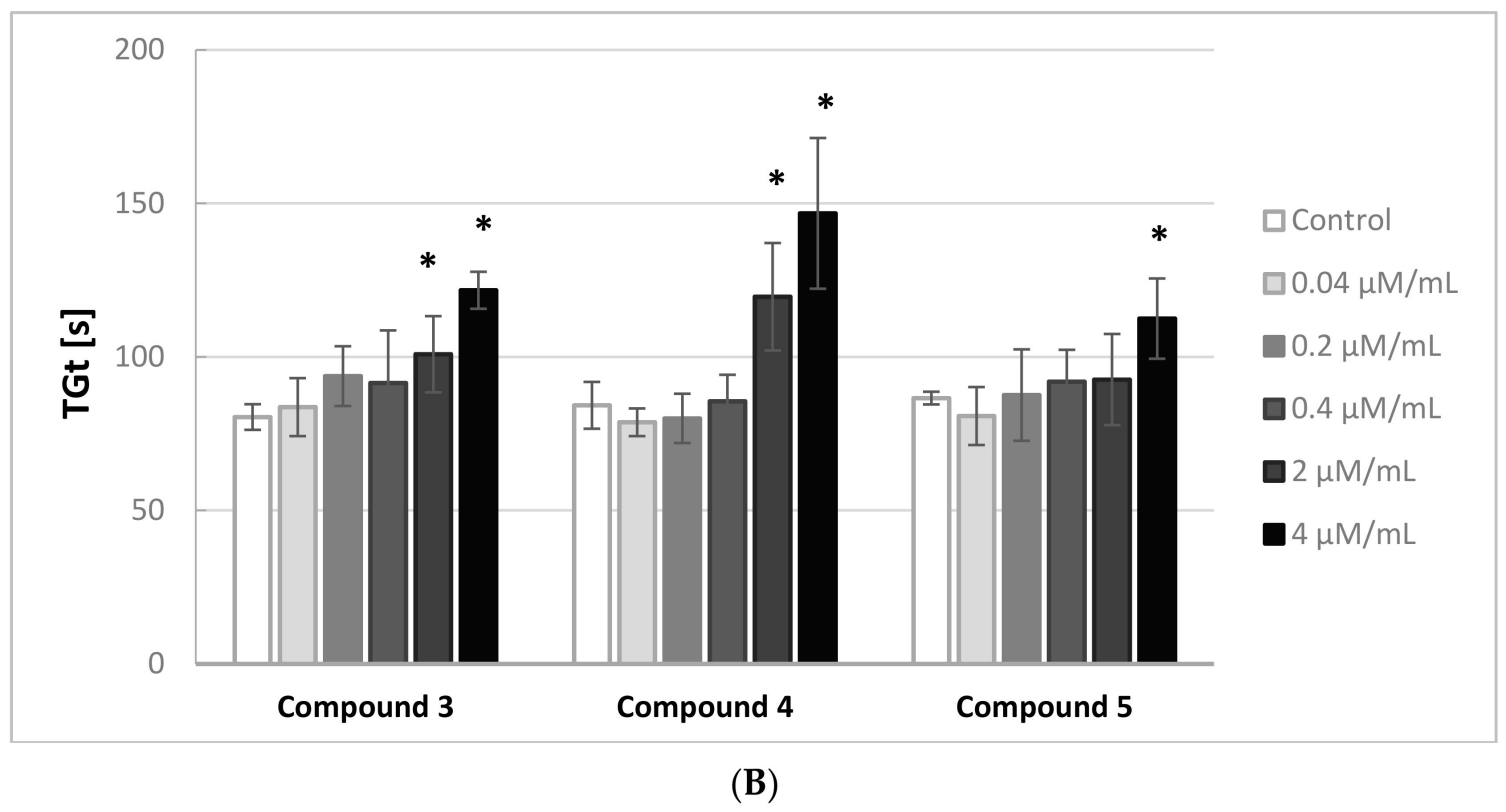
Results of coagulation assay. Influence of iminodiacetic acid derivatives (**A**) compounds **1** and **2**; (**B**) compounds **3**–**5** on thrombin generation time (TGt) (mean ± SD, *n* = 5) after 3 min incubation in plasma; final volume 500 μL. All compounds at the highest tested concentration 4 μmol/mL statistically significantly prolonged TGt. * *p* < 0.05.

Figure 2A,B illustrates the effects of the iminodiacetic acid derivatives on the thrombin generation time (TGt). Compounds **3** and **4** were shown to induce a significant increase in this parameter (↑ TGt) when added at 2.0 and 4.0 μmol/mL, whereas compounds **1**, **2** and **5** induced an increase only at the highest concentration tested.

In the case of maximum clotting (Fmax), compounds **3** and **4** contributed to the statistically significant decrease in the Fmax value at the highest concentration tested (Figure S2, Supplementary materials) This means that the structure of the tested compound determines its effect on the maximum clotting. Compounds **1**, **2** and **5** did not exert a significant effect on the value of maximum clotting (Fmax) over the entire concentration range. This lack of changes to Fmax means these compounds do not affect the structure of clot after generation of endogenous thrombin.

Depending on the compound, tested ligands at different concentrations led to the significant increase in the plasma clotting time (↑ Tf) and concomitant decrease in the initial plasma clotting velocity (↓ Fvo) (Figure S3, Supplementary materials); e.g., compounds **3** and **4** contributed to such changes at concentrations 0.4 μmol/mL and above, compounds **2** and **5** at 2.0 μmol/mL, and compound **1** only at 4.0 μmol/mL.

According to the results, compound **4** increased the area under the clot formation curve (↑ Sf) at every concentration apart from the lowest, whereas the other compounds only increased this area at the highest concentration. The observed changes to the parameters of clot formation initiated by the generation of endogenous thrombin (TGt, Fmax, Tf, Fvo) suggest that the examined ligands significantly interfered with the process only at higher concentrations. Compounds **2** and **5** were the only two that did not affect the overall potential of coagulation (S).

### 2.4. Thrombin Activity

**Figure 3:**
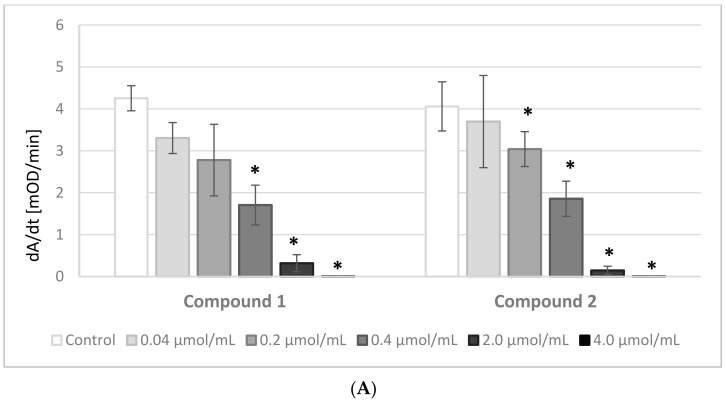

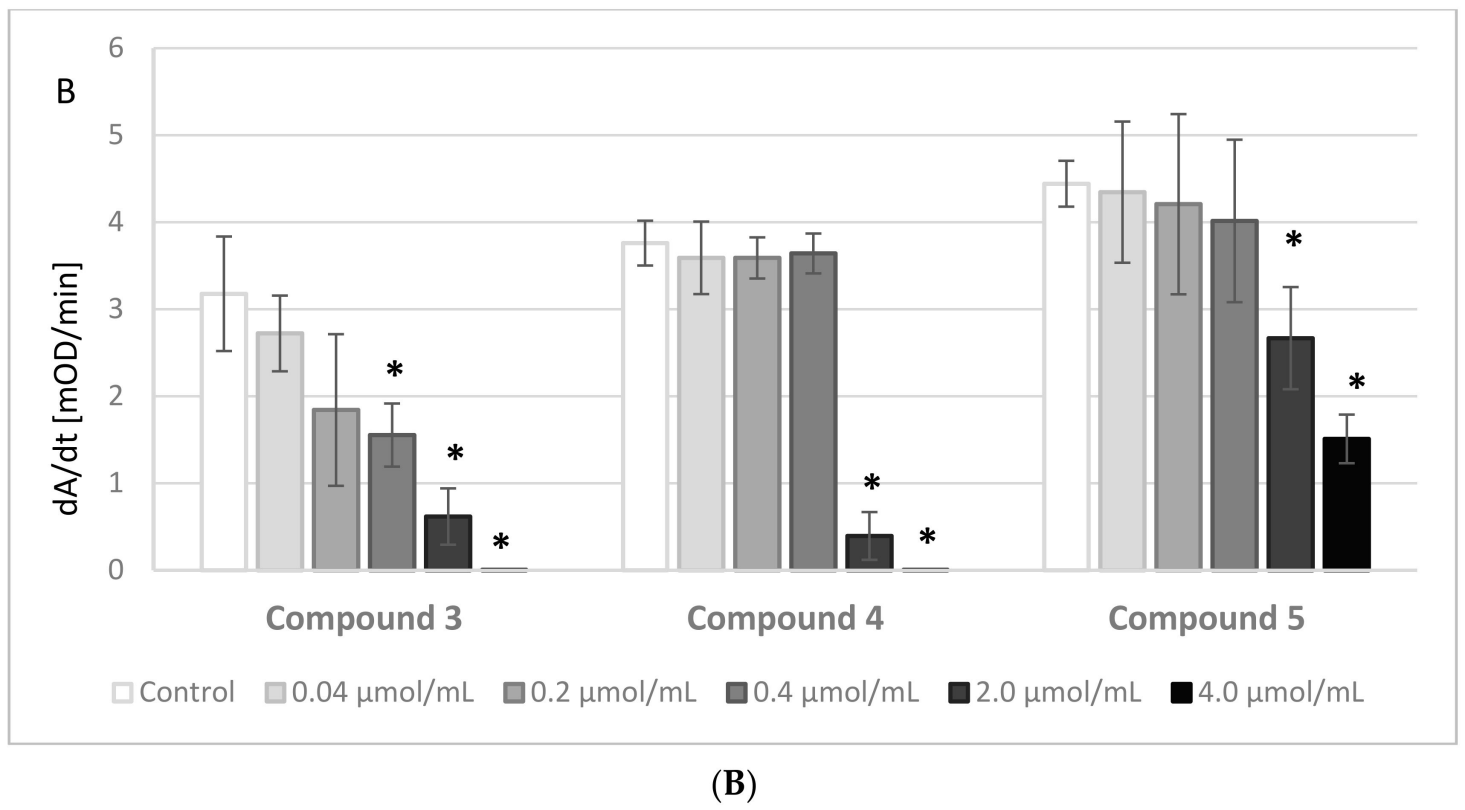
The effects of iminodiacetic acid derivatives (**A**) compounds **1** and **2**; (**B**) compounds **3–5** on amidolytic activity of thrombin expressed as velocity of the enzymatic reaction (dA/dt). The results are presented as mean ± SD, *n* = 4 after 3 min incubation in plasma; final volume 500 μL. All compounds statistically significantly decreased the velocity of the enzymatic reaction, depending on their concentration; * *p* < 0.05.

The results of the experiments evaluating the effects of IDA derivatives on the amidolytic activity of thrombin are presented in Figure 3A,B. Before the experiments, a series of tests between the analyzed compounds and the chromogenic substrate, S-2238, were performed. There were no significant changes in the absorbance of the samples; therefore, we presume that the IDA derivatives did not interact nor compete with the chromogenic substrate.

All the synthesized derivatives were examined at concentrations 0.04–4.0 μmol/mL. All compounds at the highest concentration, apart from derivative **5**, resulted in a lack of amidolytic activity of thrombin. As presented in Figure 3A,B, all compounds statistically significantly decreased the velocity of the enzymatic reaction. The results were significant at concentrations as low as 0.2 μmol/mL and higher of compounds **4** and **5**, and at even lower concentrations of the other compounds.

The maximum activity (GT_max_) corresponding to the maximum amount of p-nitroaniline released from the chromogenic substrate was determined. A statistically significant decrease in the maximum activity (GT_max_) was reported solely for 2.00 μmol/mL of IDA derivatives (Figure S4, Supplementary materials). Noticeably, compound **5** did not affect this parameter at any concentration.

### 2.5. APTT

**Figure 4:**
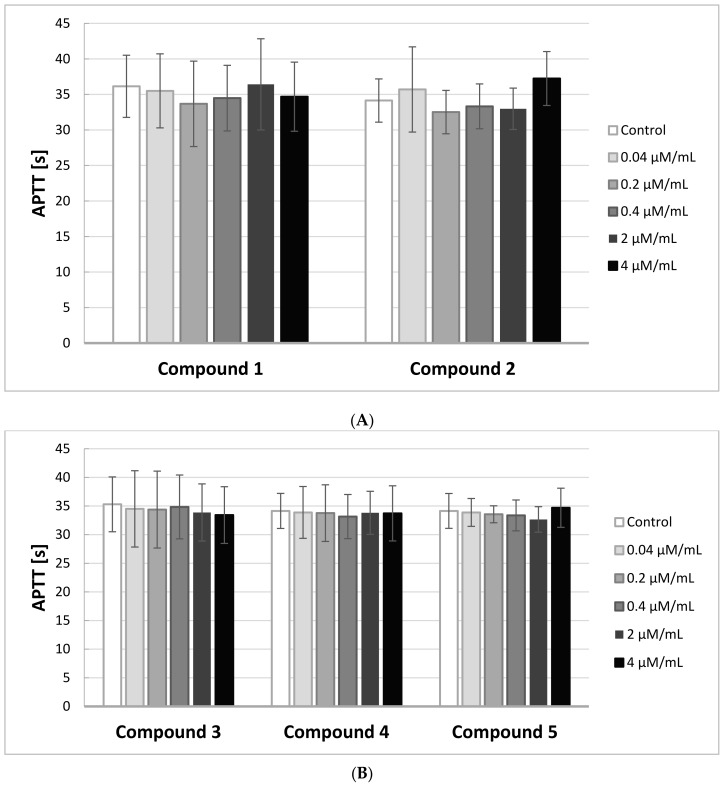
The effects of iminodiacetic acid derivatives (**A**) compounds **1** and **2**; (**B**) compounds **3**–**5** on Activated Partial Thromboplastin Time (APTT) (mean ± SD; *n* = 5) after 3 min incubation in plasma; final volume 160 μL. Exposure to the tested compounds even at the highest concentrations was shown not to significantly influence the value of APTT over the whole concentration range.

In order to identify the effects of IDA derivatives on the activity of the intrinsic and common pathways of coagulation, we performed APTT assays. The results of the experiments are illustrated on Figure 4A,B.

Any novel compound is considered biocompatible when APTT is in the range of the reference values for the method, which is between 28 and 39 s. Due to individual differences, the study was conducted on five different plasmas, coming from five different donors.

The results of the study showed that all five IDA derivatives, at all concentrations in the range 0.04–4.0 μmol/mL, did not exert any significant effect on the intrinsic coagulation pathway. All the obtained results were in the range of 28–39 s.

### 2.6. PT, INR

**Figure 5:**
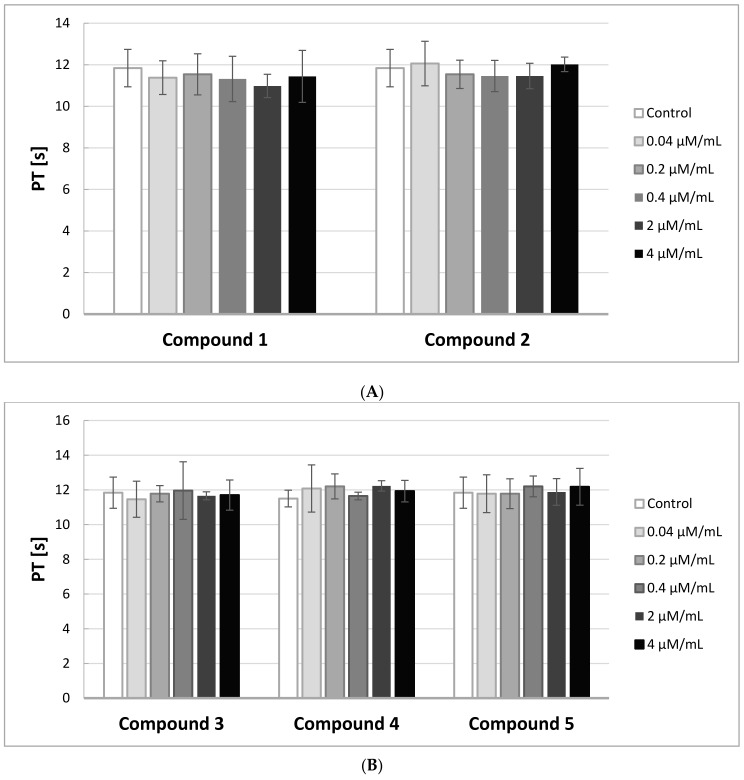
The effects of iminodiacetic acid derivatives (**A**) compounds **1** and **2**; (**B**) compounds **3**–**5** on Prothrombin Time (PT) (mean ± SD; *n* = 5) after 3 min incubation in plasma; final volume 160 μL. Iminodiacetic acid derivatives did not affect in a statistically significant way the values of PT in comparison with control.

The Prothrombin Time (PT) in contrast to the APTT gives evidence of the activity of the extrinsic and common pathways of coagulation. In Figure 5A,B, the effects of the compounds on PT are presented.

A substance is considered to be biocompatible when the coagulation time is in the range of 8.9–12.1 s, and INR between 0.9–1.2. In our study, the PT measurements were not significantly affected by the derivatives of IDA at the tested concentrations. All the obtained results were enclosed in the range of 8.9–12.1 s. Also, INR values were within the reference range.

### 2.7. TT

The Thrombin Time (TT) involves the addition of human thrombin to PPP. It, therefore, reflects the conversion of fibrinogen to fibrin. In general TT is prolonged when functional fibrinogen (FBG) levels are <1.0 g/L, however, in this paper the FBG levels were monitored and were enclosed within the reference range (2–4 g/L).

**Figure 6:**
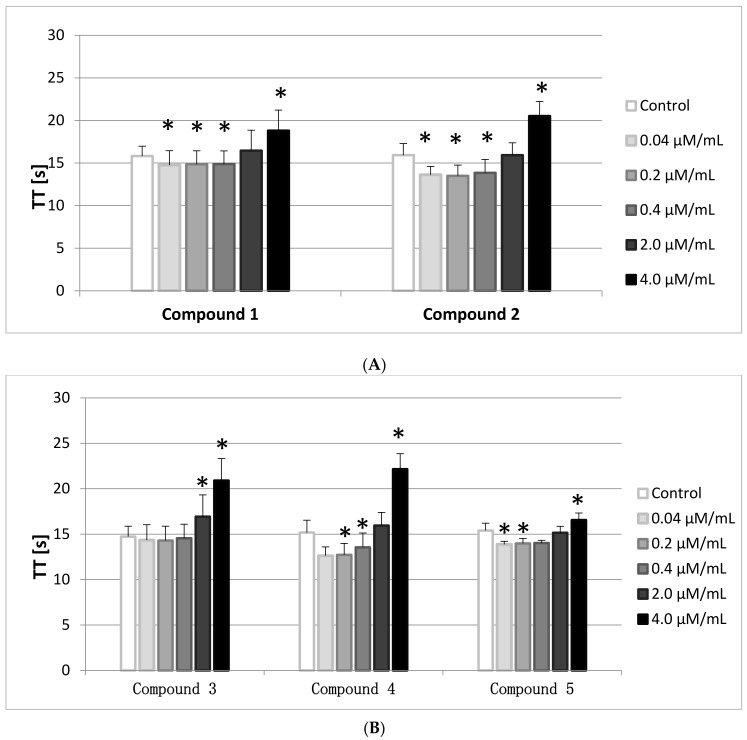
The effects of iminodiacetic acid derivatives (**A**) compounds **1** and **2**; (**B**) compounds **3**–**5** on Thrombin Time (TT) (mean ± SD; *n* = 4–5) after 2 min incubation in plasma; final volume 210 μL. All synthesized compounds apart from **3** at the lowest concentration range (0.04–0.2/0.4 μmol/mL) statistically significantly shortened TT, whereas for the highest concentration (4 μmol/mL) TT prolongation was reported. * *p* < 0.05.

The results of the conducted studies (Figure 6A,B) show that all compounds, apart from **3**, at the lowest concentration range (0.04–0.2/0.4 μmol/mL) statistically significantly shortened TT, whereas at the highest concentration (4.0 μmol/mL) TT prolongation was observed. The reference values are between 14–20 s, therefore only the results of compounds **2** and **4** did not fit within this range.

### 2.8. RBCs Lysis Assay

**Figure 7:**
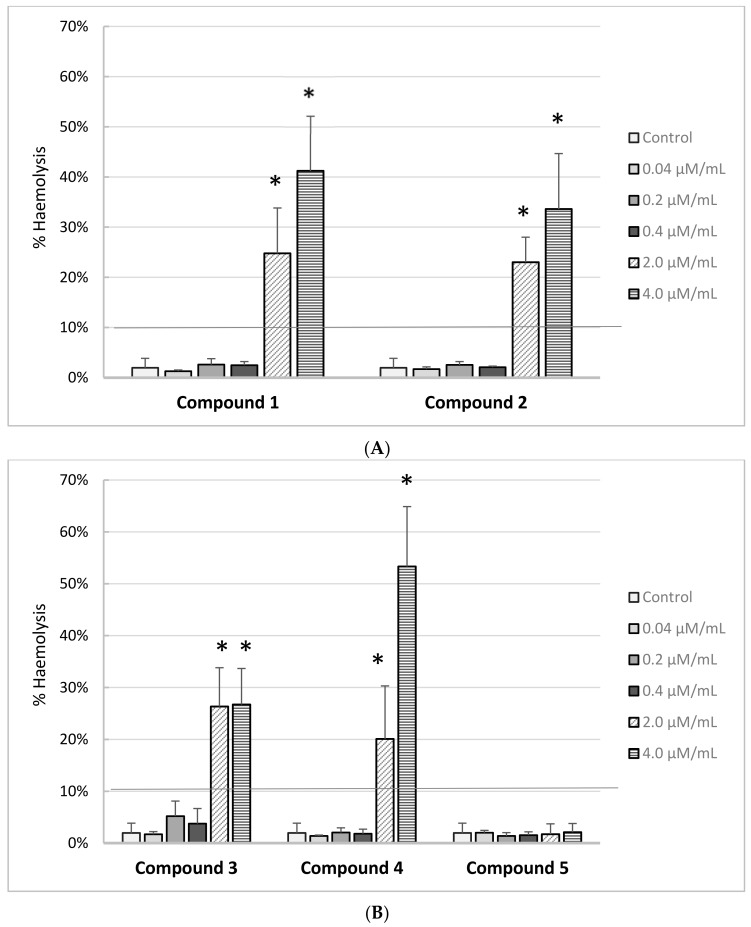
Percentage of haemolysis obtained from the interaction of iminodiacetic acid derivatives (**A**) compounds **1** and **2**; (**B**) compounds **3**–**5** with 2% RBCs (red blood cells) suspension, compared to the positive control Triton X-100 at 0.2% (100% hemolysis) (mean ± SD; *n* = 5), * *p* < 0.05 vs. control. All newly synthesized compounds showed no adverse effect on the membrane of RBCs in the concentration range 0.04–0.4 μmol/mL. A statistically significant increase in the rate of haemolysis was documented for the highest concentrations of compounds **1**–**4**.

The effects of iminodiacetic acid derivatives on the RBC haemolysis are presented on Figure 7A,B. Based on the results, we can conclude that all the newly synthesized compounds showed no adverse effects on the RBC membranes up to 0.4 μmol/mL corresponding to the theoretical plasma concentration. The percentage of haemolysis exceeded 10% for compounds **1**–**4** at concentrations of 2.0 μmol/mL and 4.0 μmol/mL. This indicates that these compounds at concentrations exceeding 2 μmol/mL may exert adverse effects on the membranes of other cells, which may be important for further tests using in vitro cell cultures. Only derivative **5** showed complete biocompatibility over the entire concentration range.

### 2.9. RBCs Morphology

**Figure 8:**
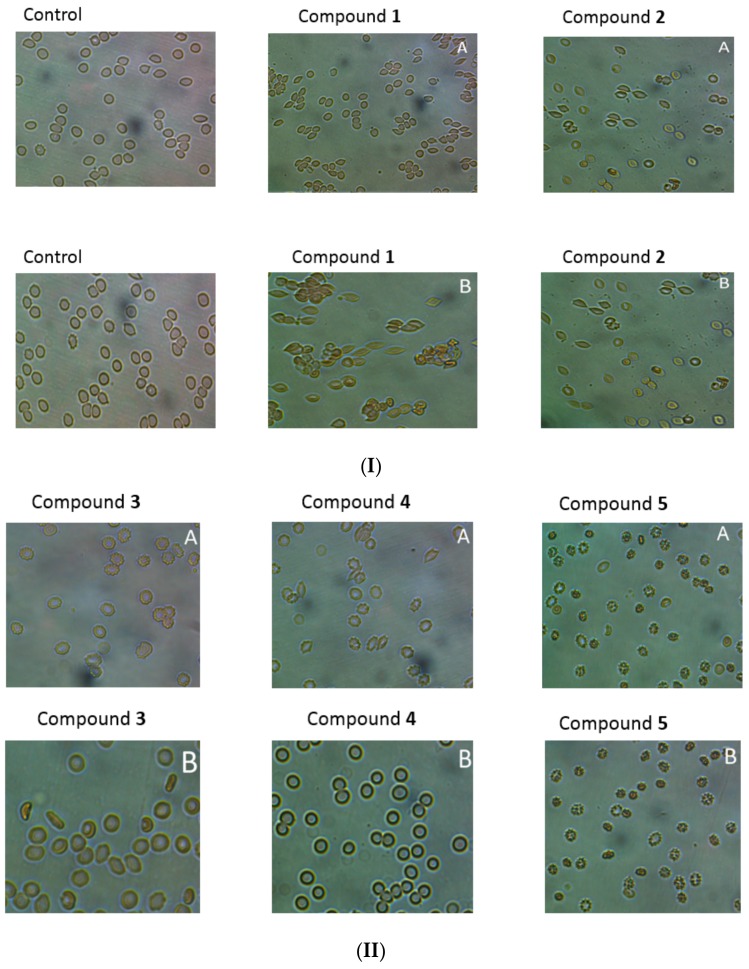
(**I**) Effect of iminodiacetic acid derivatives on erythrocytes morphology. 2% erythrocyte suspension was treated at 37 °C for 60 min with indicated concentrations of compound **1** and **2**. Representative phase-contrast images are shown (magnification of 400 times). A—concentration 0.2 μmol/mL; B—concentration 2.0 μmol/mL; (**II**). Effect of iminodiacetic acid derivatives on erythrocytes morphology. 2% erythrocyte suspension was treated at 37 °C for 60 min with indicated concentrations of compound **3**–**5**. Representative phase-contrast images are shown (magnification of 400 times). A—concentration 0.2 μmol/mL; B—concentration 2.0 μmol/mL.

Microscopic evaluation of the suspension of erythrocytes and the tested compounds after incubation (37 °C, 1 h), showed, depending on the compound and concentration, tendency for stomatocyte and echinocyte formation. It was found that the tendency to form echinocytes and stomatocytes, as well as hemolysis of erythrocytes, depends on the concentration of the compound in the environment. As shown in Figure 8I,II, incubation of erythrocytes with compound **1** at the concentration of 0.2 μmol/mL resulted in the formation of single stomatocytes, whereas in the case of higher concentrations (2.0 μmol/mL) the percentage of stomatocytes was significant. For compound **2**, we observed a tendency to form stomatocytes (Figure 8I,II), but also single eryptotic erythrocytes were distinguished. Derivatives **3** and **4** at the lower concentrations (0.2 μmol/mL) contributed to the formation of echinocytes. On the other hand, incubation with these compounds at a concentration of 2.0 μmol/mL resulted in anisocytosis, characterised by erythrocytes of varying size and shape. In the microscopic image, large ovalocytes can be seen explicitly (Figure 8). The changes observed in the higher concentrations correspond to RBCs membrane damage observed in the lysis study. Compound **5** had a strong tendency for eryptosis at both high and low concentrations. Haemolysis was not reported at any concentration of compound **5**; therefore, it might be concluded that this compound leads to programmed death of RBCs.

### 2.10. RBCs Flow Cytometry Studies

**Figure 9:**
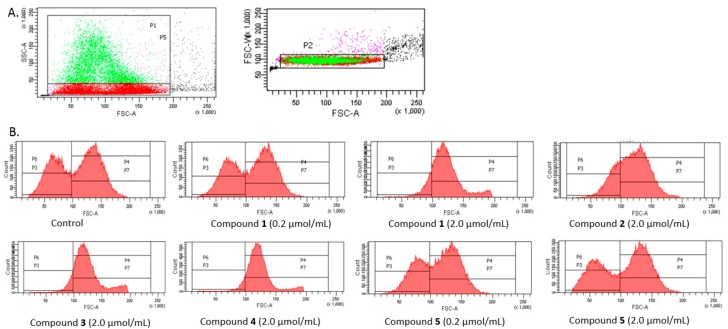
Effects of iminodiacetic acid derivatives on erythrocytes (flow cytometry studies). (**A**) On the basis of SSC-A (side scattered light A) parameter, subpopulation of erythrocytes marked in grey and separated by gate P5 was distinguished; (**B**) Bimodal distribution of RBCs—control and samples treated with IDA derivatives (0.2 and 2.0 μmol/mL). Erythrocytes were divided into P3 and P4 gates differing in the value of FSC (Forware Scatter) parameter corresponding to the size of measured objects. The erythrocytes of gate P5 were divided into P6 and P7 according to FSC parameter.

The analysis was conducted on a few separated gates (Figure 9A). On the basis of SSC-A (side scattered light A), an indicator of cell granularity or internal complexity, one subpopulation marked as light grey and separated by gate P5 was segregated from all analysed erythrocytes. In the case of controls, this population constituted approximately 20.1 ± 0.91% of all erythrocytes. Among all measured events, the erythrocytes were divided into P3 and P4 gates varying with regard to the FSC (Forware Scatter) parameter value (corresponding to the size of the measured objects). Additionally, the erythrocytes of gate P5 were divided into P6 and P7 according to the FSC parameter.

**Figure 10:**
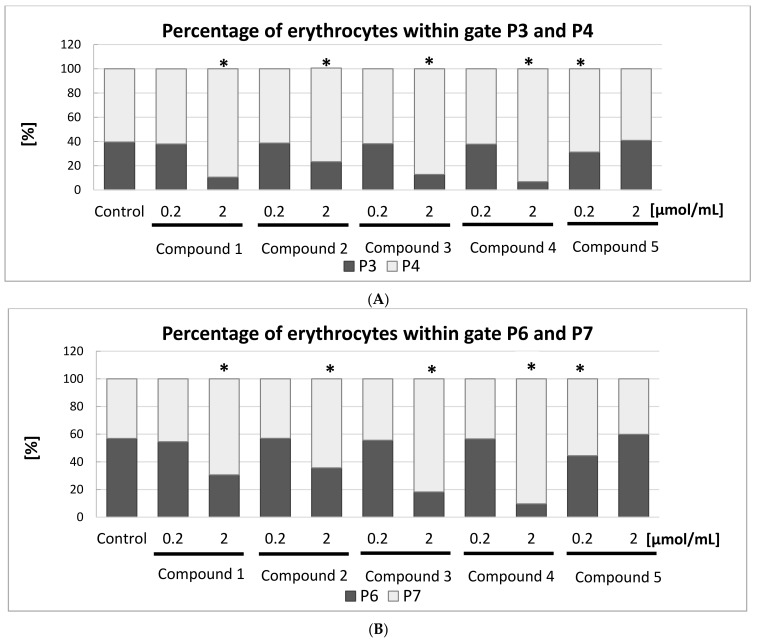
Effects of iminodiacetic acid derivatives on erythrocytes. (**A**) Percentage of all RBCs (mean ± SD, *n* = 3) divided into P3 and P4 gates. The highest tested concentrations (2.0 μmol/mL) of compounds **1**–**4** and compound **5** at concentration of 0.2 μmol/mL contributed to significant changes in the amount of RBCs within these gates; (**B**) Percentage of all RBCs divided into P6 and P7 gates. The highest tested concentrations (2.0 μmol/mL) of compounds **1**–**4** contributed to significant changes in the amount of RBCs within these gates. * *p* < 0.05 vs. control.

As presented in Figure 9B, FSC (Forware Scatter) histograms show a typically bimodal distribution of RBCs which corresponds to the ellipsoid, biconcave RBCs (control). The percentage of RBCs in gates P3 and P4 and P6 and P7, for the samples treated with the five IDA derivatives at two concentrations are presented in Figure 10A,B.

Figure 10A depicts the percentage of all RBCs divided into gates P3 and P4. The statistical analysis found all the IDA derivatives, apart from compound **5**, at the highest concentration tested contributed to significant changes in the amount of RBCs within these gates. At lower examined concentrations, compound **5** caused a significantly lower amount of RBCs within the gate P3. These results are also confirmed by the histograms presented in Figure 9B: a typical bimodal distribution of RBCs can be seen for lower concentrations of compound **1** and 2.0 μmol/mL of compound **5**, whereas compounds **1**–**4** at a concentration of 2.0 μmol/mL contributed to the significant changes in the distribution of RBCs.

This study also showed that compounds **1**–**4** induced significant changes in the morphology of RBC subpopulation P5 (Figure 10B). For instance, the percentage of RBCs in the P6 gate decreased from 56.9% to 9.6% after one hour of incubation with compound **4** at a concentration of 2.0 μmol/mL, which might be due to the destruction of the erythrocyte membrane. Inversely, the percentage of RBCs in gate P7 increased from 43.1% to 90.4%.

## 3. Discussion

The primary physiological response to any chemical substance including contrast agents (CAs) administered intravenously involves the interaction with blood components and usually sustains unwanted targets. CAs used for MRI may interact with various biological systems, including the coagulation system, blood platelets and plasma proteins, which may lead to serious clinical implications [26]. Taking into consideration the above-mentioned literature examples and the necessity to establish the biocompatibility of potential ligands for technetium complexation, the purpose of this study was to assess the effect of five novel iminodiacetic acid derivatives on plasma haemostasis by measuring the overall potential of clot formation and fibrinolysis and the kinetic parameters of these processes, and to draw the conclusion which group of iminodiacetic acid derivatives presents favourable properties regarding plasma haemostasis and effects on morphology of RBCs.

In CL-test, the formation of fibrin was induced by high concentrations of exogenous thrombin [27,28]. Thrombin time (Tt) in this test is the time elapsed from the addition of agonist (thrombin) to the beginning of clot formation. Regarding this parameter, we observed a complex impact of novel IDA derivatives. At the lower concentrations, the tested ligands shortened Tt (↓ Tt), whereas at the highest concentration significant prolongation of Tt (↑ Tt) was observed. These results were mostly confirmed by the measurements of Thrombin Time (TT), a routinely used laboratory test. All these experiments were conducted under stable conditions, with constant concentration of exogenous thrombin and unaltered fibrinogen (FBG) concentration. We assumed that the changes in thrombin time could be a result of the compounds influence on the amidolytic activity of the enzyme. On contrary, our previous work [15] has shown that IDA derivatives with methyl substituents in aromatic ring and mebrofenin significantly prolonged Tt in CL-test without its shortening at lower concentrations.

The results regarding Tt in CL-test attempted us to conduct further studies and evaluate the effects of IDA derivatives on enzymatic activity of thrombin. The results confirmed that all the IDA derivatives significantly decreased the initial velocity of the reaction when added at concentrations of 0.4 μmol/mL and higher, depending on the studied compound. Substantially, at the highest concentration (4.0 μmol/mL), all ligands apart from **5**, contributed to the disappearance of enzymatic activity of thrombin. These results were further confirmed by the maximum activity (GTmax) which at the highest concentration of compounds **1**–**4** was so low that it could hardly be estimated (Figure S4, Supplementary materials). On the basis of these results, we presume that the shortened thrombin time reported for lower concentrations of ligands might be caused by their influence on the polymerization of fibrin.

As all experiments were conducted under the stable concentration of FBG, the documented changes in the Fmax value for the highest tested concentration (↓ Fmax) might be attributed to the changes in the structure and arrangement of the clot. Similarly to our previous study [15], all the newly synthesized IDA derivatives, depending on their concentration, caused a significant increase in the plasma clotting time (↑ Tf) and concomitant decrease in the initial plasma clotting velocity (↓ Fvo). To sum up, the profile of changes in the parameters of clot formation suggest that the examined ligands markedly impaired the process of coagulation when added at high concentrations. Ligand **5** appears to interfere with this process at the lowest rate.

With regard to the phase of clot stabilization, we reported that compounds **1**, **3** and **5** did not contribute to changes in clot stabilization time (Tc) over the entire concentration range. Only compound **2** at 4.0 μmol/mL and compound **4** at 2.0 μmol/mL significantly prolonged Tc (↑ Tc), which could have clinical implications as an increase in Tc may lead to a delay in the process of fibrinolysis and prolong the time elapsed between the initiation of the clotting and complete lysis of the clot [27,28]. These results are important especially in view of the other publications reporting fibrinolysis resistance of clots formed in the presence of mebrofenin [15].

In the case of fibrinolysis, it should be highlighted that all reported changes in maximum fibrinolysis (Lmax) stem from changes in maximum clotting (Fmax). It is a crucial result, as it demonstrates the complete lysis of a previously formed clot. Regarding the kinetic parameters of the fibrinolysis process, compound **3** did not exert a significant effect on the initial velocity of fibrinolysis (Lvo), whereas the other compounds, depending on the concentration, significantly decreased its value (↓ Lvo). In our previous paper [15] we found that only derivative with 2 methyl groups in aromatic ring and mebrofenin at the concentration of 4.0 μmol/mL contributed to the significant decrease in Lvo. However, further studies on the effect of IDA derivatives on plasmin activity should be performed to elucidate the mechanism of its influence on the process of fibrinolysis.

To sum up, we should highlight that despite the significant influence of the tested compounds on the kinetic parameters of each evaluated process, the overall potential of clot formation and fibrinolysis (CL_AUC_) remained unchanged for most compounds. However, in the case of compound **1**, it was decreased. This observation is crucial in view of our previous results [15] where methyl derivatives were associated with significant increase in the overall potential of clot formation and fibrinolysis (↑ CL_AUC_).

During the experiment evaluating the influence of novel IDA derivatives, similarly to methyl derivatives [15], on the generation of endogenous thrombin, we observed a significant increase in the thrombin generation time (↑ TGt) for the highest concentration tested. Similarly to the CL-test, the calculations of Fvo showed a concentration dependent decrease of this value with simultaneous increase in coagulation time (↑ Tf). These effects suggest that tested ligands may alter the activity of coagulation factors.

Coagulation can be triggered either by surface-mediated reactions (intrinsic coagulation pathway), or by exposure to factors derived from damaged tissue (extrinsic coagulation pathway). These two routes join into a common path leading to the formation of a fibrin clot [29]. For the evaluation of the effects of IDA derivatives on these two coagulation pathways, Prothrombin Time (PT) and Activated Partial Thromboplastin Time (APTT) were performed. Considering both these tests, we did not report any statistically significant effects of the new IDA derivatives at any concentration in the range: 0.04–4.0 μmol/mL. All the obtained results were enclosed in the reference values; this indicates the tested compounds are potentially biocompatible.

Besides the interactions with materials and plasma proteins, a series of interactions with blood cells might be initiated. Within this realm, biocompatibility refers to the quantification of cellular and plasma components of the blood. Apart from the biocompatibility aspect, we cannot omit the role of erythrocytes in the coagulation process [30]. Pretorius et al. [31] indicated that erythrocytes are extremely sensitive cells and form a crucial component of the human body as health indicators. Generally, hemocompatibility is evaluated through haemolysis of RBCs, which is considered a simple and reliable measurement for estimating blood biocompatibility [32]. Within this study we found that compounds **1**–**4** at concentrations of 2.0 μmol/mL and higher contribute to haemolysis exceeding 10%. This indicates that these compounds may exert an adverse effect on the membranes of RBCs. However, potential systemic effects of synthesized IDA derivatives on the erythrocyte haemolysis should be regarded as clinically irrelevant. The haemolysis exceeding 10% was observed mostly at 2.00 and 4.00 μmol/mL, which is about 5–10-fold higher than the theoretical plasma concentration of these compounds. When comparing to the previous study, we can conclude that all IDA derivatives, excluding derivative **5**, exhibit comparable effect on the permeability of erythrocytes membrane, as the clinically significant hemolysis was reported at the concentration of 2.00 and 4.00 μmol/mL.

Light microscopy studies together with flow cytometry experiments enabled the effect of IDA derivatives on the morphology of RBCs to be evaluated. All compounds, apart from **5**, contributed to the formation of either stomatocytes or echinocytes. Shape conversion of erythrocytes from the most physiological—biconcave—in stomatocytes or echinocytes depends on many factors including pH, ionic strength, and the interaction of xenobiotics [33]. Many drugs have been found to affect the shape of the RBCs via interaction with components of erythrocyte membranes, such as lipids and proteins. Changes in the shape of erythrocytes after stimulation with xenobiotics have been proposed to arise from the differential expansion of the two monolayers of the membrane lipid bilayer. According to Sheetz and Singer [34], echinocytes are formed during the insertion of xenobiotics into the outer monolayer.

The microscopy analysis of compound **5**, the only one that did not cause haemolysis at any concentration, revealed a high percentage of RBCs eryptosis manifested by cell shrinkage and membrane scrambling. Eryptosis is a process of programmed cell death and might be caused by hyperosmotic shock, energy reduction, or inflammatory markers that lead to oxidative stress (e.g., protein C, coagulation factors, prostaglandin E2 etc.) [35,36]. Apart from physiological and pathological factors, eryptosis might be caused by a number of drugs [35,36]. According to Lang [35], eryptosis is responsible for the removal of defective erythrocytes, the prevention of haemolysis and the release of concomitant haemoglobin. There are also studies reporting that some drugs contributing to eryptosis may favourably influence the clinical course of malaria by clearing infected erythrocytes from circulating blood [35]. On the other hand, drug-induced eryptosis may lead to erythropenia and anemia. Furthermore, eryptotic erythrocytes may also stimulate blood clotting, which, as a consequence, can impede microcirculation [36].

The results of the heamolysis assays and microscopy studies, as well as the flow cytometry experiments, provide the full picture of the influence of IDA derivatives on the integrity of RBC membranes and morphology.

## 4. Materials and Methods

### 4.1. Materials

The substrates for the synthesis of iminodiacetic acid derivatives, namely nitrilotriacetic acid (cat. No. N9877), m-anisidine (cat. No. A8820-4), 2-methoxy-5-methylaniline (cat. No. 103284), 2,4-dimethoxyaniline (cat. No. D129801), 3,4-dimethoxyaniline (cat. No. A8300-8), and 3,5-dimethoxyaniline (cat. No. D13000-1), were purchased from Sigma Aldrich (Poznan, Poland). Acetic anhydride (cat. No. 242845) and pyridine (cat. No. 270970) were provided by Sigma Aldrich and the solvents, i.e., methyl alcohol (cat. No. 621990110), ethyl alcohol (cat. No. 396480427), dichloromethane (cat. No. 628410421), sodium hydroxide (cat. No. 810981118) and hydrochloric acid (cat. No. 575283115) by Polish Chemical Reagents (Gliwice, Poland). All the chemicals were used without further purification.

We used thrombin produced by Biomed (Kraków, Poland) and recombinant tissue plasminogen activator (t-PA) manufactured by Boehringer-Ingelheim (Ingelheim am Rhein, Germany). Tris-buffered saline (TBS, cat. No. SRE0032) was purchased from Sigma Aldrich. Calcium chloride (cat. No. 875010112) and sodium chloride (cat. No. 794121116) were provided by Polish Chemical Reagents. Triton X-100 (cat. No. 841810492) was obtained from Polish Chemical Reagents. For APTT assays we used Bio-Ksel System APTTs reagent and calcium chloride (Bio-Ksel, Grudziądz, Poland). Bio-Ksel PT plus reagent (tromboplastin and solvent) was used in PT tests, while thrombin (3.0 UNIH/mL, Bio-Ksel, Poland) was utilized in TT tests.

### 4.2. Synthesis of N-(Acetanilide)-Iminodiacetic Acid Derivatives

Iminodiacetic acid derivatives were synthesized as described previously [8]. pKa values were calculated according to available software [37]. Briefly, *N*-(3-methoxy-acetanilide)iminodiacetic acid (MW = 296.31 g/mol; p*K*_a_ = 2.8; compound **1**), *N*-(2-methoxy-5-methyl-acetanilide)iminodiacetic acid (MW = 310.34 g/mol; p*K*_a_ = 3.1; compound **2**), *N*-(2,4-dimethoxy-acetanilide)iminodiacetic acid (MW = 326.34 g/mol; p*K*_a_ = 2.7; compound **3**), *N*-(3,4-dimethoxy-acetanilide)iminodiacetic acid (MW = 326.34 g/mol; p*K*_a_ = 2.6; compound **4**) and *N*-(3,5-dimethoxy-acetanilide)iminodiacetic acid (MW = 326.34 g/mol; p*K*_a_ = 2.5; compound **5**) were obtained during reactions between the in situ obtained nitrilotriacetic acid anhydride and appropriate aniline derivative (Figure S1, Supplementary materials).

The structures of the obtained compounds were verified based on ^1^H-NMR, ^13^C-NMR, elemental analysis and IR spectra. Melting points were estimated with an Electrothermal apparatus (all data presented in Supplementary Materials). The synthesized compounds were of chromatographic grade purity. The logP values for the IDA derivatives were calculated with a ChemSketch program: *N*-(3-methoxyacetanilide)iminodiacetic acid (**1**) (logP = 0.64 ± 0.49), *N*-(2-methoxy-5-methylacetanilide)-iminodiacetic acid (**2**) (logP = 0.83 ± 0.48), *N*-(2,4-dimethoxyacetanilide)iminodiacetic acid (**3**) (logP = 0.26 ± 0.49), *N*-(3,4-dimethoxy-acetanilide)iminodiacetic acid (**4**) (logP = 0.56 ± 0.51) and *N*-(3,5-dimethoxyacetanilide)-iminodiacetic acid (**5**) (logP = 0.69 ± 0.53).

### 4.3. In Silico Structure-Activity Evaluation

For basic structure-activity studies, the free online Molinspiration tool was used: www.molinspiration.com. For prediction of physico-chemical properties https://ilab.acdlabs.com/iLab2/index.php [25] was used.

### 4.4. Plasma Preparation for CL-Test, APTT, PT, TT Measurements

The studies on biological material were approved by the Bioethics Committee of the Medical University of Lodz, Poland (RNN/109/16/KE).

Blood samples were obtained from healthy donors from the Blood Donation Centre in Lodz, Poland. The blood was collected in vacuum tubes containing 3.2% buffered sodium citrate. Poor platelet plasma (PPP) was obtained by centrifugation (3000× *g*, 20 min, room temperature) with a Micro 22R centrifuge (Hettich Zentrifugen, Tuttlingen, Germany). Small portions of PPP were stored up to one month at −30 °C. Before the studies, each sample of PPP was restored in a water bath at 37 °C for 15 min. Once thawed, the PPP was not frozen again nor used for retesting.

### 4.5. Test of Clot Formation and Lysis (CL-Test)

The CL-test evaluated the global assays of coagulation and fibrinolysis by continuous measurement of the optical transmittance alterations. It established the influence of the tested compounds on the overall potential of clot formation and fibrinolysis, as well as their kinetic parameters [27,38]. The method used was a modification of the optical measurement of coagulation and blood fibrinolysis, presented by Glover et al. [39] and He et al. [40,41].

General conditions of the experiments were presented previously [15]. Briefly, the measurements were taken at λ = 405 nm on a spectrophotometer Cecil CE 2021 (Cecil Instruments Ltd., Cambridge, UK) at 37 °C. Tested compounds at the appropriate concentrations and t-PA (220 ng/mL) were added to diluted plasma, and the samples were incubated at 37 °C for three minutes. Afterwards, thrombin (0.5 IU/mL) was added to initiate clot formation.

Prior to the experiments, we performed reagent tests in order to check whether the synthesized compounds form precipitates with human plasma. No changes in both the absorbance and visual precipitation were observed following the ten-minute incubation of the tested compounds with the plasma.

The obtained curves of clot formation and fibrinolysis were analyzed using dedicated software [38]. This program estimated general parameters of the examined process: the overall potential of clot formation and fibrinolysis (CL_AUC_), which is the area under clotting, fibrinolysis and transition curves, and total time of clot formation and fibrinolysis (T). Additionally, the kinetic parameters of the processes of clot formation and fibrinolysis were counted. (I) clot formation phase: Tt—thrombin time, Fmax—maximum clotting, Tf—plasma clotting time, Fvo—initial plasma clotting velocity, Sf—area under the clot formation curve; (II) clot stabilization phase: Tc—clot stabilization time, Sc—area under the curve of stable clot formation; (III) fibrinolysis: Lmax—maximum lysis, Tl—fibrinolysis time, Lvo—initial clot fibrinolysis velocity, Sl—area under the fibrinolysis curve.

The method of clot formation and fibrinolysis was validated, and the coefficient of variation (W) for single human plasma (*n* = 4) was within the range 1.9–8.3% depending on the calculated parameter.

### 4.6. Coagulation Assay

General conditions of coagulation assay were described elsewhere [15,27]. The experiments were conducted at λ = 405 nm, by means of a thermostatic spectrophotometer (Cecil CE 2021). The synthesized compounds at five concentrations (in a 10 μL volume) were added to plasma diluted three times with TBS buffer, and the samples were incubated at 37 °C for 3 min. Then a mixture of thrombin (0.0375 IU/mL) and calcium chloride (0.015 mmol/mL) was added.

The obtained curves were analysed by means of a software [38]. The following kinetic parameters were established: TGt—thrombin generation time, Fmax—maximum clotting, Tf—plasma clotting time, Fvo—initial plasma clotting velocity, Sf—area under the clot formation curve, Sc—area under the clot stabilization time, and S—area under the curve of coagulation.

The method of clot formation and fibrinolysis was validated, and the coefficient of variation (W) for single human plasma (*n* = 4) was within the range 1.6–7.7% depending on the parameter.

### 4.7. APTT

APTT assays were performed on coagulometer CoagChrom-3003 Bio-Ksel according to the available commercial method. PPP with APTT reagent and the tested compound or water (control) was incubated at 37 °C for 3 min. Afterwards, the addition of calcium chloride initiated clotting. The APTT measured the time it took for fibrin clot formation after the addition of calcium chloride.

Validation of the method conducted on Bio-Ksel plasma: Normal and Abnormal plasma revealed that the coefficient of variability was as follows: W = 0.75% for Normal plasma (Bio-Ksel), W = 2.49% for Abnormal plasma (Bio-Ksel).

### 4.8. PT, INR

PT tests were conducted on coagulometer (CoagChrom-3003, Bio-Ksel) according to the commercially available method. PPP was incubated with 10 μL of the tested compound or water (control) at 37 °C for one minute. Afterwards, the PT reagent (thromboplastin) was added. PT time was recorded as the time taken for clot formation after the addition of the reagent.

Validation of the method was conducted on Bio-Ksel plasma Normal and Abnormal plasma. The coefficient of variability was counted (W = 2.56% for Normal plasma, W = 4.10% for Abnormal plasma).

### 4.9. Thrombin Time (TT)

TT assays were performed according to the available commercial method (coagulometer CoagChrom-3003 Bio-Ksel). PPP with the tested compound or water (control) was incubated at 37 °C for 1 min. Afterwards, the addition of 100 μL of thrombin (3.0 UNIH/mL) initiated clotting. Coefficient of variability (W) for the method was 1.02%.

### 4.10. Thrombin Activity

The amidolytic activity of thrombin was determined using chromogenic substrate D-Phe-Pip-Arg-pNA as previously described [15]. All the experiments were conducted in TBS buffer (pH = 7.4) with a final volume of 500 μL. Thrombin (0.112 IU/mL) and iminodiacetic acid derivatives at concentrations of 0.04 μmol/mL to 4.00 μmol/mL, or appropriate volume of TBS (control), were added to a cuvette and incubated for 3 min at 37 °C. 20 μL of 2 mmol/L chromogenic substrate was added to start the reaction, and then continuous absorbance measurements were taken at λ = 405 nm wavelength with a spectrophotometer (Cecil CE2021) at 37 °C for 10 min.

The obtained curves were evaluated using dedicated computer software. The following parameters were estimated: dA/dt—initial velocity of reaction and GT_max_—maximum activity.

### 4.11. RBC Lysis Assay

The RBCs lysis assays were performed with the blood obtained from the Blood Donation Centre in Lodz. The blood from healthy donors was collected into tubes containing a solution of potassium EDTA. RBCs were separated from the plasma by centrifugation (3000 × *g*, 10 min) at 20 °C and washed three times with 0.9% saline.

The influence of the iminodiacetic acid derivatives on the integrity RBC membranes was evaluated with the method described elsewhere [15]. Briefly, RBCs suspension (2% in NaCl) was incubated at 37 °C with the tested compounds at appropriate concentrations. After a 1-h incubation, the samples were centrifuged at 1000× *g* for 10 min and the absorbance of the supernatant was measured at 550 nm. The degree of haemolysis was expressed as a percentage of released haemoglobin. Positive (Triton X-100 (2.0% *v*/*v*)) and negative controls (saline solution) were used [15]. The coefficient of variability was counted (W = 9.51%, *n* = 5).

### 4.12. Microscopy Studies

Two percent erythrocyte suspension was incubated at 37 °C for 60 min with tested compounds at concentrations 0.04–4 μmol/mL. After this time, the suspension was diluted two-fold on titration plates. RBCs morphology was observed using the phase contrast Opta-Tech inverted microscope (magnification of 400 times), equipped with software (OptaView 7, Opta-Tech, Warsaw, Poland) for image analysis.

### 4.13. Flow Cytometry-SC Parameter

The size of the RBCs after incubation with iminodiacetic acid derivatives was assessed by a flow cytometry (FMC) technique performed on FACS Canto II (Becton Dickinson, Franklin Lakes, NJ, USA). FMC gate on erythrocytes was established for data acquisition and the cell sizes were determined with low angle light scattering. The data were displayed in diagrams of the cell number versus the light scattered. The study was limited to 20,000 events and lasted 20–30 s. The tested compounds at concentrations of 0.2 μmol/mL and 2.0 μmol/mL were added to RBCs suspension, prepared the same way as in the RBCs lysis test, and incubated for 1 h at 37 °C. Afterwards the samples were diluted 4-fold and analysed by a flow cytometer.

### 4.14. Statistics

Statistical analysis was conducted with a commercially available package (Statistica 12.0, StatSoft, Krakow, Poland). All results are presented as means ± SD. Normal distributions of continuous variables were verified with the Shapiro-Wilk test. Paired *t*-tests were used for intergroup comparisons of normally distributed variables, while the variables with non-normal distributions were compared using the Wilcoxon signed rank test. The results of all the tests were considered significant at *p*-values lower than 0.05.

## 5. Conclusions

The current study presents the synthesis of new iminodiacetic acid derivatives, and their effects on the processes of clot formation and fibrinolysis, as well as on the integrity and morphology of erythrocytes.

Despite their significant influence on the kinetic parameters of the processes of clot formation and fibrinolysis, the overall potential of clot formation and lysis (CL_AUC_) was not altered by ligands 3 and 5. The measurements of the total time of the process (T) showed the statistically significant prolongation of this parameter, however, these changes were reported only at the highest concentrations of the compounds. Coagulation assay revealed that high concentrations of the synthesized compounds contributed to a prolonged thrombin generation time (TGt) and a decreased initial velocity of coagulation. This was further explained by the inhibition of the amidolytic activity of thrombin.

Both routine laboratory methods, PT and APTT, estimating the extrinsic and intrinsic coagulation pathways, respectively, confirmed the biocompatibility of the tested compounds; all obtained results were enclosed within the reference values. The profile of changes reported for methoxy derivatives appears to be similar to IDA derivatives containing methyl substituents, however, some differences could be distinguished. For instance, methoxy derivatives affect the process of fibrin polymerization which was reflected by shortened TT.

On the basis of the haemolysis assays, we conclude that all newly synthesized compounds showed no adverse effects on the membrane of RBCs within the concentration range 0.04–0.4 μmol/mL. Compound **5** did not increase the haemolysis rate at any concentration. Furthermore, the microscopy studies revealed eryptotic changes in the RBCs treated with this ligand. Other synthesized ligands contributed to the formation of physiological forms of RBCs (echinocytes and stomatocytes).

Despite the documented effects the synthesized IDA derivatives have on plasma haemostasis and RBCs, they should generally be considered safe for use. Their significant effects were observed mostly at 4.00 μmol/mL, which is about 10-fold higher than the theoretical plasma concentration of these compounds. Among all synthesized IDA derivatives, both with methyl [15] and methoxy substituents in aromatic ring, the most promising candidate for further studies appears to be compound **5** possesing 2 methoxy groups at position 3 and 5 in benzene ring.

## Figures and Tables

**Table 1:** Molinspiration-calculated properties of iminodiacetic acid derivatives.

Properties	Compound 1	Compound 2	Compound 3	Compound 4	Compound 5
milogP	−1.40	−1.39	−1.78	−2.17	−1.78
TPSA	106.93	116.17	125.40	125.40	125.40
Number of atoms	20	22	23	23	23
MW	280.28	310.31	326.31	326.31	326.31
nON	7	8	9	9	9
nOHNH	3	3	3	3	3
n violations	0	0	0	0	0
nrotb	7	8	9	9	9
Volume	249.18	274.73	283.71	283.71	283.71

milogP—theoretically calculated logP; TPSA—Total Polar Surface Area; MW—Molecular Weight; nON—number of hydrogen bond acceptors; nOHNH—number of hydrogen bond donors; n violations—number of violated drug-likeness rules; nrotb—number of rotating bonds, Volume—molecular volume.

**Table 2:** Physicochemical characterization of iminodiacetic acid derivatives.

Properties	Compound 1	Compound 2	Compound 3	Compound 4	Compound 5
Molar Refractivity (cm^3^)	72.58 ± 0.3	77.41 ± 0.3	79.26 ± 0.3	79.26 ± 0.3	79.26 ± 0.3
Parachor (cm^3)^	596.0 ± 4.0	633.0 ± 4.0	652.7 ± 4.0	652.7 ± 4.0	652.7 ± 4.0
Index of Refraction	1.611 ± 0.02	1.603 ± 0.02	1.595 ± 0.02	1.595 ± 0.02	1.595 ± 0.02
Surface tension (dyne/cm)	66.1 ± 3.0	62.5 ± 3.0	61.5 ± 3.0	61.5 ± 3.0	61.5 ± 3.0
Density (g/cm^3^)	1.417 ± 0.06	1.377 ± 0.06	1.400 ± 0.06	1.400 ± 0.06	1.400 ± 0.06
Polarizability (×10^−24^ cm^3^)	28.77 ± 0.5	30.68 ± 0.5	31.42 ± 0.5	31.42 ± 0.5	31.42 ± 0.5

**Table 3:** The effects of synthesized iminodiacetic acid derivatives on the kinetic parameters of clot formation and fibrinolysis (CL-test).

Parameters								
Comp.	Conc. (μmol/mL)	Tt (s)	Fmax (%T)	Fvo (%T/min)	Tc (s)	Lvo (%T/min)	CL_AUC_ (%T·min)	T (s)
**1**	Control	39.6 ± 4.52	72.3 ± 7.30	158.2 ± 15.73	212.3 ± 17.94	31.9 ± 3.38	426.4 ± 69.81	498.1 ± 29.65
	0.04	38.1 ± 3.96	72.8 ± 7.44	154.8 ± 18.13	196.2 ± 23.36	32.1 ± 3.62	389.6 ± 71.75	473.9 ± 49.10
	0.2	39.0 ± 3.37	71.3 ± 8.30	**133.8 ± 6.55**	195.5 ± 24.2	38.3 ± 2.95	**361.43 ± 88.49**	500.4 ± 32.74
	0.4	39.6 ± 2.05	69.9 ± 7.04	**125.0 ± 11.85**	185.3 ± 22.59	28.5 ± 3.78	**375.4 ± 85.96**	477.3 ± 41.29
	2.0	44.7 ± 3.43	**62.7 ± 5.72**	**101.3 ± 17.36**	200.2 ± 13.38	**23.7 ± 4.54**	**357.7 ± 73.34**	525.2 ± 28.02
	4.0	**64.13 ± 10.19**	**64.7 ± 5.66**	**58.3 ± 16.95**	207.8 ± 14.58	**21.42 ± 6.41**	**327.4 ± 90.07**	**679.8 ± 89.88**
**2**	Control	46.7 ± 8.09	64.3 ± 11.64	121.9 ± 22.65	205.0 ± 60.69	27.9 ± 7.74	370.0 ± 152.98	496.2 ± 122.11
	0.04	**10.7 ± 1.53**	65.3 ± 8.49	**65.1 ± 9.48**	172.2 ± 32.39	21.6 ± 5.21	380.6 ± 97.48	488.9 ± 105.24
	0.2	**18.3 ± 5.57**	**43.0 ± 13.85**	**24.9 ± 13.70**	**125.7 ± 32.34**	**12.1 ± 3.77**	**237.9 ± 109.27**	525.4 ± 108.52
	0.4	**16.5 ± 11.60**	58.6 ± 14.4	**60.7 ± 36.85**	187.8 ± 76.07	**17.6 ± 4.01**	366.2 ± 168.46	**526.2 ± 114.04**
	2.0	**24.3 ± 14.71**	60.1 ± 15.98	**67.1 ± 17.47**	218.7 ± 67.98	**17.9 ± 5.51**	450.5 ± 213.76	**604.3 ± 138.91**
	4.0	55.5 ± 16.47	50.5 ± 14.68	**38.8 ± 21.69**	**282.3 ± 88.86**	21.3 ± 7.51	403.6 ± 223.84	**807.8 ± 126.72**
**3**	Control	41.2 ± 5.71	67.3 ± 10.70	150.4 ± 24.06	203.3 ± 29.46	28.8 ± 5.48	378.1 ± 99.82	480.3 ± 42.44
	0.04	40.5 ± 6.62	67.9 ± 10.07	**114.9 ± 31,53**	183.7 ± 30.23	30.0 ± 2.74	336.0 ± 72.91	480.6 ± 55.06
	0.2	38.9 ± 9.13	63.9 ± 10.43	**95.9 ± 19.48**	177.4 ± 23.28	26.9 ± 4.46	328.2 ± 98.48	492.6 ± 44.19
	0.4	43.6 ± 5.35	65.73 ± 10.21	**91.2 ± 18.35**	184.7 ± 23.75	27.4 ± 3.64	351.4 ± 97.44	498.8 ± 59.48
	2.0	**53.7 ± 11.12**	61.29 ± 10.72	**69.2 ± 24.94**	199.7 ± 27.14	23.5 ± 5.55	371.4 ± 111.08	539.6 ± 46.75
	4.0	86.1 ± 18.10	**41.6 ± 13.34**	**44.5 ± 11.73**	223.4 ± 38.45	19.5 ± 7.87	309.6 ± 105.37	**732.4 ± 75.06**
**4**	Control	36.2 ± 5.9	73.2 ± 10.69	169.5 ± 30.67	283.5 ± 83.51	24.9 ± 8.63	527.1 ± 123.12	608.8 ± 167.29
	0.04	**18.0 ± 5.01**	70.5 ± 15.21	**85.9 ± 45.85**	**210.0 ± 46.96**	20.2 ± 9.86	499.6 ± 119.8	633.1 ± 137.53
	0.2	**21.5 ± 4.72**	66.1 ± 20.09	**93.8 ± 68.72**	**200.2 ± 84.63**	**19.9 ± 6.96**	472.1 ± 162.43	602.4 ± 158.91
	0.4	**19.2 ± 6.31**	70.5 ± 14.01	**109.1 ± 98.85**	249.0 ± 106.28	**18.9 ± 10.40**	483.1 ± 229.27	638.9 ± 198.52
	2.0	35.9 ± 5.98	51.3 ± 6.65	**127.4 ± 49.57**	**347.2 ± 113.51**	23.8 ± 11.08	**679.9 ± 188.31**	**773.4 ± 262.66**
	4.0	33.9 ± 10.09	**78.5 ± 18.83**	**33.3 ± 15.38**	310.7 ± 96.15	**11.5 ± 7.55**	513.6 ± 108.85	**965.4 ± 214.35**
**5**	Control	35.8 ± 2.69	66.0 ± 6.57	137.5 ± 15.14	200.5 ± 29.75	25.1 ± 3.93	386.9 ± 68.86	481.5 ± 57.57
	0.04	27.4 ± 10.52	64.0 ± 7.85	114.1 ± 47.83	194.6 ± 75.77	23.4 ± 5.94	389.3 ± 110.95	514.9 ± 80.39
	0.2	34.4 ± 5.33	60.6 ± 12.95	120.8 ± 38.03	201.3 ± 28.40	21.9 ± 4.56	371.2 ± 91.64	508.0 ± 48.06
	0.4	36.8 ± 4.96	64.4 ± 10.30	116.3 ± 34.31	217.3 ± 49.21	23.3 ± 5.79	408.9 ± 96.99	524.0 ± 79.96
	2.0	49.7 ± 21.91	64.3 ± 9.56	111.8 ± 57.87	241.0 ± 79.39	23.6 ± 3.95	436.1 ± 140.56	**563.2 ± 71.07**
	4.0	39.7 ± 9.31	**52.1 ± 9.69**	**65.9 ± 29.75**	248.0 ± 73.69	**14.9 ± 2.72**	397.4 ± 33.27	**671.4 ± 106.98**

The results are given as mean ± SD; *n* = 7–8. Values marked in bold are statistically significant (*p* < 0.05). Tt—thrombin time, Fmax—maximum clotting, Fvo—initial plasma clotting velocity, Tc—clot stabilization time, Lvo—initial clot fibrinolysis velocity, CL_AUC_—the overall potential of clot formation and fibrinolysis, T—total time of the process of clot formation and fibrinolysis.

## Supplementary Materials

Supplementary materials are available online.
